# Analysis of Epstein-Barr virus infection models in a series of pediatric carriers from a developing country

**DOI:** 10.1038/srep23303

**Published:** 2016-03-18

**Authors:** Aldana G. Vistarop, Melina Cohen, Elena De Matteo, María Victoria Preciado, Paola A. Chabay

**Affiliations:** 1 Molecular Biology Laboratory, Pathology Division, Ricardo Gutiérrez Children’s Hospital, Buenos Aires, Argentina; 2 Pathology Division, Ricardo Gutiérrez Children’s Hospital, Buenos Aires, Argentina

## Abstract

Epstein-Barr virus (EBV) is a B lymphotropic human herpesvirus. Two models, germinal center (GC) and direct infection, describe how EBV infects B-cells. Since in Argentina primary infection is mostly subclinical at young ages, children represent an interesting population where to analyze EBV infection, especially considering that most studies are usually performed in adults. Tonsil biopsies from pediatric carriers were studied to describe infection characteristics. EBV+ lymphocytes at the interfollicular region were mainly observed. Latency III pattern in subepithelial (SubEp) lymphocytes was observed at young ages, probably indicating a recent infection. In older patients EBV was mostly detected in epithelial cells, suggesting that they could have been infected some time ago. This finding was sustained by tonsillar viral load, which was higher in cases with LMP1+SubEp cells vs. LMP1+nonSubEp cells (p = 0.0237, Mann-Whiney test). Latency III was prevalent and related to the GC, while latency II was associated with non-GC (p = 0.0159, χ2 test). EBERs+/IgD+ cells were statistically prevalent over EBERs+/CD27+ cells (p = 0.0021, χ2 test). These findings indicated that both EBV infection models are not mutually exclusive and provide some basis for further understanding of EBV infection dynamics. Moreover, we provide a more accurate explanation of EBV infection in pediatric asymptomatic carriers from a developing country.

Epstein-Barr virus (EBV) is acquired early in life and establishes an asymptomatic infection of the B lymphoid system. The delicate EBV-host balance could be disturbed, consequently the virus could display its pathogenic potential; nevertheless, the reasons why EBV is involved on the development of tumors are still under discussion^1^,^2^. The main target cells for EBV are B lymphocytes; nonetheless, the virus is also able to infect and transform epithelial cells^3^. EBV is linked to Burkitt Lymphoma (BL), Hodgkin Lymphoma (HL), some types of non-Hodgkin’s lymphoma, a subset of T and NK cell lymphomas and nasopharyngeal carcinoma (NPC)^2^,^3^.

EBV-associated tumors display three classical patterns with differential expression of latency proteins, in part as a consequence of host immune response to EBV antigens. Latency (L) III expresses EBNA1, -2, -3A, -3B, -3C, -LP, together with LMP1 and -2, and the non-coding RNAs (EBERs, microRNAs, and BARTs) and is typically associated with post-transplant lymphoproliferative disorders. All other latency types involve increasing degrees of viral gene silencing, namely LI, observed in BL, expresses only EBNA1 and the non-coding RNAs, and LII related to HL displays EBNA1, LMP1 and -2 and the non-coding RNAs^4^.

After primary infection, EBV resides mainly in the long-lived memory B-cells, but how the virus reaches there could be explained by two different proposed infection models. The “germinal center” model postulates that the initial infection of naïve B-cells drives their proliferation and the expression of LIII pattern. Then EBV-infected B-cells enter the germinal center (GC) and express LII program, and finally exit from GC as EBV+ memory B-cells expressing Latency 0 program (L0), with complete viral gene silencing to avoid recognition by EBV-specific immune cells^5^. Eventually, EBV-infected dividing memory B-cells can switch to the LI pattern, where EBNA1 allows the viral episome to be segregated into daughter cells. Initially, through *in vitro* studies, LMP1 and LMP2A were proposed to rescue pro-apoptotic B-cells at GC^6^. However, a modest signaling role for LMP1 and LMP2A in GC B-cells was demonstrated *in vivo*, based on the presence of high affinity antibodies in LMP1+ and -2A+ B-cells^7^. The “direct infection model” suggests that EBV directly infects memory B-cells, drives them to proliferate, without taking part at the GC reaction^8^. In line with this, LMP1+ B-cells at the interfollicular (IF) region were described in infectious mononucleosis (IM) adult cases, and an incompatibility of LMP1 expression with the entry of the infected cells into the GC was suggested^9^.

How EBV gains access to the B-cell compartment is still under debate. On one hand, efficient production of virus in stratified epithelium, which expressed latency-associated EBV proteins in addition to lytic-cycle viral proteins, was demonstrated *in vitro*, but a population of epithelial cells that exclusively expressed latency-associated viral proteins could not be detected^10^. On the other hand, it has been demonstrated *in vitro* that EBV transmigrates across oral epithelial cells without causing productive infection^11^; thus, the virus might get into the B cell compartment in the tonsillar crypts by crossing the thin layer of epithelium overlying the lymphocytes below^12^.

The age at primary infection varies substantially worldwide since exposure to EBV is likely to be affected by socioeconomic factors. In developing countries, primary infection occurs during childhood, disseminates via saliva and is largely asymptomatic. However, in developed populations, primary infection can be delayed until the second decade of life or even later, and can manifest itself as IM in approximately 25–75% of EBV-infected persons^1^. It is important to remark that there are differences between children and adolescent regarding the immune response^13^. One hallmark of IM is global CD8+ T-cell lymphocytosis, mostly reflecting a huge expansion of activated EBV-specific CD8+ T-cells^14^. In contrast, it was recently described in African children that asymptomatic EBV infection elicits a specific CD8+ T-cell response that can control the infection without over-expansion^14^. Moreover, it was suggested that preexisting NK cell populations found in children may provide an explanation for why IM occurs more frequently in adolescents and adults than in children^15^,^16^. In Argentina, nearly 70% of patients are seropositive by the age of 2 years, like in other developing countries. In addition, EBV association with patients younger than 10 years described in several types of pediatric B-cell lymphomas may suggest a close relationship between low age of EBV seroconversion and increasing risk of B-cell lymphoma development^17^. Therefore, our pediatric population shows interesting epidemiological EBV infection characteristics to study viral pathogenesis.

The aim of this study was to characterize in tonsils of pediatric carriers, the histological regions where viral antigen expression is located and to distinguish the B cell population infected by EBV, in order to shed some light on EBV infection models taking place at the main site of infection.

## Results

### EBV latent and lytic protein expression

**Figure 1:**
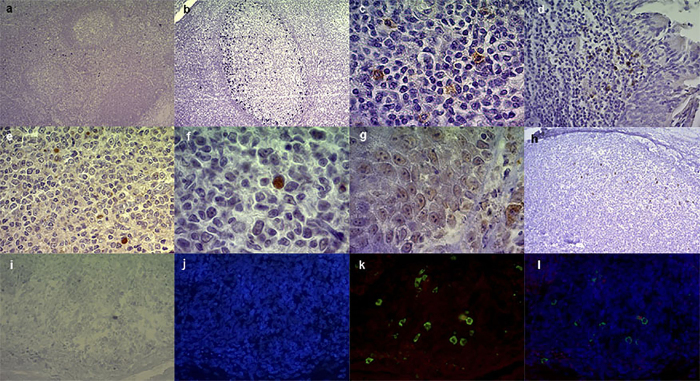
Histological location of viral antigens and EBERs/CD27 double immunostaining cells in tonsil sections from pediatrics carriers. (**a**) EBERs-specific *in situ* hybridization with RNA probes revealed specific black-dark blue nuclear staining at the IF region (10X). (**b**) Numerous EBERs positive cells within the GC region (20X). (**c**) LMP1 (100X), and (**d**) LMP2A (40X) membranous and cytoplasmic positive staining at the IF region and subepithelial lymphocytes, respectively. (**e**) EBNA2 (40X) and (**f**) EBNA3A (100X) nuclear positive staining at the IF and GC region respectively. (**g**) Subepithelial EBNA3A positive macrophages (cytoplasmic) and epithelial cells (nuclear) staining (100X). (**h**) Nuclear staining of BMRF1 positive cells at the IF region (10X). (**i**) EBERs-specific *in situ* hybridization with RNA probes reveals EBV-positive cells (black-dark blue labelling) changed false red color to do the merge and built EBERs/CD27 double immunostaining image. (**j**) Counterstaining with fluorescent nucleus Hoechst; (**k**) goat anti-mouse FITC antibody to detect mouse anti-CD27 (green); (**l**) merged images. Digital images were obtained with an AxioCamErc 5s (Zeiss) camera and acquired using Digital AxioVision Rel. 4.8 image acquisition software.

EBERs transcripts and EBV protein expression profile defining viral latency patterns, in relation to age and histological characteristics are summarized in Table 1. EBV antigens staining by IHC are shown in Fig. 1a–h.

EBV latent proteins LMP1, LMP2A, EBNA2 and 3A and the BMRF1 lytic antigen were studied in each case. LMP1 expression localized to the cytoplasm and surface membrane of cells was detected in all cases, whereas LMP2A was identified in 19/29 cases. In addition, EBNA2 and EBNA3A were observed at the cell nucleus in 19/29 and 10/29 cases, respectively. Finally, only 5/29 cases displayed the nuclear BMRF1 lytic antigen. The integrated analysis of viral antigen expression was performed to discriminate latency patterns, which were identified as follows: LI, cases with EBERs expression; LII, EBERs together with LMP1; LIII, LII proteins together with EBNA2 expression. Based on this scenario, 10/29 cases displayed LII, whereas 19/29 exhibited LIII pattern. LI and 0 were not found.

**Figure 2:**
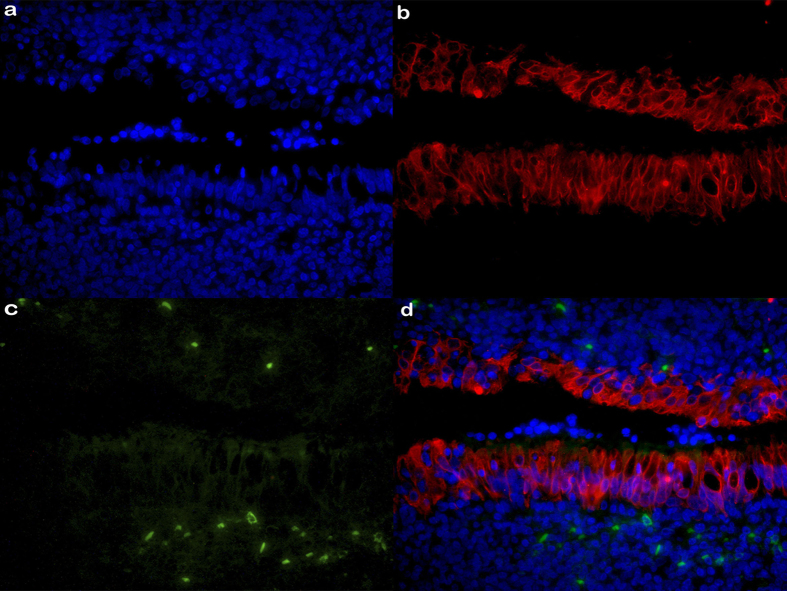
LMP2A - CK7 double immunostaining cells in tonsil sections from pediatrics carriers. (**a**) Counterstaining with fluorescent nucleus Hoechst; (**b**) goat anti-rabbit AlexaFluor®568 antibody to detect rabbit anti-CK (red); (**c**) rabbit anti-rat FITC antibody to detect rat anti-LMP2A (green) and (**d**) merged images (40X). Digital images were obtained with an AxioCamErc 5s (Zeiss) camera and acquired using Digital AxioVision Rel. 4.8 image acquisition software.

With regards to histological location, we characterized viral antigen expression in lymphocytes at GC, interfollicular (IF) and subepithelial (SubEp) regions, as well as in epithelial cells (Table 1). Most viral antigens studied were located at the IF region. Particularly, EBERs expression was located exclusively at the IF region in 22/29 cases while it was restricted solely to the GC region in two of them. The remaining 5 cases displayed EBERs+ cells at both regions. LMP1 expression prevailed at IF or IF-SubEp regions, whereas only 1 case displayed LMP1 expression at both IF and GC. BMRF1+ cells were located exclusively at IF region in all cases. LMP2A+ cells were almost restricted to IF regions in 18/19 cases, which included some few cases showing LMP2A staining also at SubEp area. Concerning EBNA3A+ cells, IF location was predominant; just two cases showed specific SubEp EBNA3A+ cells and some cases showed GC location. Finally, EBNA2+ lymphocytes were distributed at random at IF, SubEp and/or GC regions in all 19 EBNA2+ samples. Interestingly, some cases showed specific LMP2A+ (Fig. 2), EBNA3A+, BMRF1+ and/or EBNA2+ epithelial cells.

When LII and LIII were compared in relation to its location within the GC vs. non GC, unexpectedly, we found that LII was statistically associated with non GC, while LIII was prevalent within the GC (p = 0.0159, χ2 test).

We remarkably observed positive staining of EBNA3A, LMP1 and EBNA2 in macrophages in certain cases. Particularly, 6 samples from EBV+ carriers exhibited EBNA3A positive staining exclusively in SubEp macrophages cytoplasm, probably representative of phagocytosis. Since the expected lymphocyte staining was not observed, we did not assume these cases as EBNA3A+. Regarding EBNA3A antigen detection in macrophages, it was prevalent in the group of older children, with a mean age of 11 years (p = 0.0193, Mann Whitney test).

**Figure 3:**
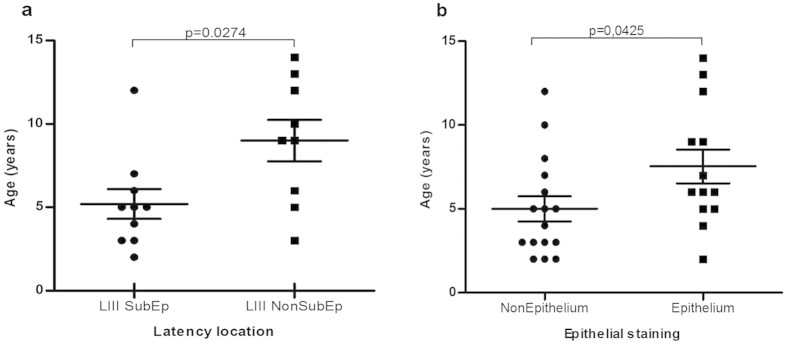
Comparison of patient’s means age according to EBV antigens location. Patient’s age distribution analyzed according to the observed location of EBV antigens in (**a**) LIII SubEp *vs*. LIII non SubEp, and (**b**) Epithelium *vs*. non Epithelium (p = 0.0274 and p = 0.0425, respectively; Mann Whitney test).

In order to deeply characterize viral location and its relationship with patients’ age, we initially compared mean age of cases with EBV antigen expression at the SubEp region vs. outside the SubEp region and a trend to lower mean age was found in the former group (p = 0.0683, Mann Whitney test). Remarkably, when only the LIII pattern was considered, cases with antigen expression in SubEp lymphocytes were significantly younger than those without them (4 vs. 9 years; p = 0.0274, Mann Whitney test) (Fig. 3a). Moreover, those cases with LMP1+ lymphocytes at the SubEp were statistically younger than those exclusively depicting staining at the IF region (4 vs. 7 years; p = 0.0483, Mann Whitney test).

We also analyzed the mean age of those patients whose biopsies showed epithelial cells with positive staining for EBNA2, EBNA3A, LMP2A and BMRF1 and compared them with their negative counterpart. It was noticed that positive cases were older than the negative ones, with a statistically significant difference (8 vs. 5 years; p = 0.0425, Mann Whitney test) (Fig. 3b).This finding was confirmed when analyzing exclusively LIII cases, since those with positive epithelial cells were significantly older than those lacking them (9 vs. 5 years; p = 0.0127, Mann Whitney test).

### EBV viral load in tonsil samples

With the purpose of characterize to a greater extent EBV infection in pediatric carriers from a developing population; EBV viral load was assessed in tonsil samples. Detected viral loads ranged from 30 to 372 copies/μg DNA. No correlation was found between EBV copy number and patients’ age (p > 0.05, Spearman correlation test). In line with this, no statistical association was observed in median viral load between cases with lytic antigen BMRF1 expression and their negative counterpart (p > 0.05, Mann Whitney test). When median viral load was analyzed in relation to latency profiles, viral copy number was the same when latency II and III patterns were compared (median 60 copies/μg DNA; p = 0.05, Mann Whitney test).

Remarkably, when cases with LMP1+ SubEp cells were analyzed, a higher median copy number than those without LMP1 in SubEp cells was depicted (p = 0.0237, Mann Whitney test).

### Characterization of EBV infected cells

**Figure 4:**
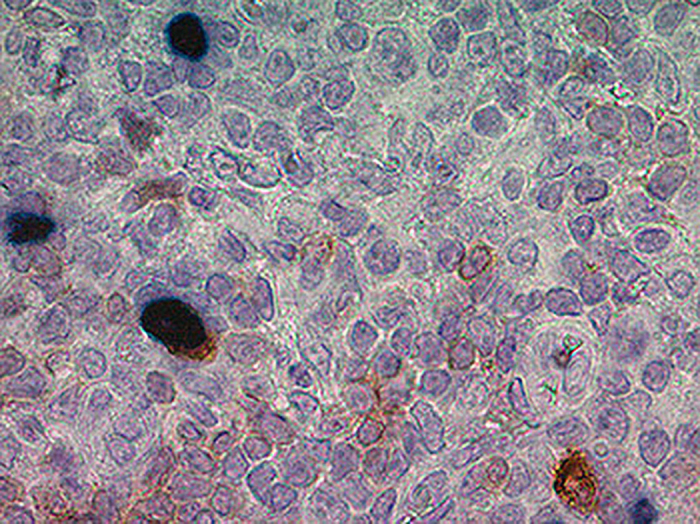
EBERs - IgD double immunostaining cells in tonsil sections of pediatrics carriers. The figure shows representative expression of a EBERs+ cell, an IgD+ cell and a EBERs+/IgD+ cell. EBV+ cells (nuclear black-dark blue staining) were determined by EBERs ISH and IgD positive cells (membrane brown staining) were assessed by IHC. All tissues were counterstained with haematoxylin and digital images were obtained with an AxioCamErc 5s (Zeiss) camera and acquired using Digital AxioVision Rel. 4.8 image acquisition software.

With the aim of deeply characterize EBV infected cells and describe EBV infection models in our series, we performed double staining for EBERs and CD20 (B-cell marker), CD27 (memory B-cell marker) or IgD (naïve cell marker or memory B-cell without isotype switch). Twenty two out of 29 cases were positive for both EBERs and CD20 marker. Whereas 4/29 and 15/29 cases showed EBERs with CD27 or IgD positive cells, respectively (Figs 1i–l and 4). In addition, 3/4 EBERs+/CD27+ cases exhibited EBERs+/IgD+ cells as well. Remarkably, cases with EBERs+/IgD+ cells were statistically prevalent in our pediatric carriers (p = 0.0021, χ2 test). Patients with EBERs+/CD27+ cells exhibited a trend to lower median age than those without them (p = 0.0692, Mann Whitney test), whereas no statistical differences in median age were observed among patients with EBERs+/IgD+ cells (p = 0.2648, Mann Whitney test). Neither EBERs+/CD27+ nor EBERs+/IgD+ cases showed a specific association with LII or III patterns histological location (p = 0.3724, χ2 test). Finally, viral load in patients with EBERs+/IgD+ cells showed a trend to higher values (p = 0.0576, Mann Whitney test).

## Discussion

EBV infection models were never explored in recently infected pediatric carriers from a developing country, so, our series represents an interesting group to study the first steps of EBV infection. In this study, 29 EBV+ tonsil biopsies from pediatric carriers were analyzed. First of all, we assessed EBERs expression, and find out that it was mainly observed in lymphocytes from IF region, as previously described by Hudnall, *et al*.^18^. Then, in order to assess if EBV+ cells were naïve or memory B-cells, EBERs plus IgD or CD27 staining was performed. Double-stainings revealed that IgD+/EBERs+ cells prevailed over CD27+/EBERs+ ones. EBV expression on IgD+ cells was previously described by Chaganti *et al*., who detected viral DNA in the IgD+/CD27- naïve B-cells and in the IgD+/CD27+ non-switched memory B-cell fraction from both IM patients and chronic carriers, but in contrast with our observations, EBV viral loads were considerably lower than the determined in samples with switched memory cells^19^. Therefore, our results support the notion that EBV is able to infect both naïve B-cells and non-switched memory B-cells. In addition, given that we found EBERs+/CD27+ cells only in 4 patients, and 3 of them also had EBERs+/IgD+ cells, EBV presence in memory B-cells could be a consequence either of direct infection, or of a previous naïve infected B-cell which maturated to memory B-cell. Finally, although CD20+/EBERs+ cells were predominantly detected in almost all cases, double staining failed to be detected in 7 of them, perhaps because other cells, such as T or NK cells, were the infected ones as previously observed^18^.

On the contrary to the “GC model”, LMP1 expression in our patients was observed exclusively outside the GC, and not always accompanied by LMP2A expression. This fact could imply that LMP1+ B-cells had already left the GC, but were still expressing the protein, or that they had never passed throughout this region. Furthermore, LMP2A signal was proposed to restore normal GC development and to allow LMP1-expressing B-cells to enter GC^20^. This could explain that the lack of LMP2A in LMP1+/LMP2A- cases prevented LMP1+ cells to get into the GC.

EBV antigen expression was assessed in order to discriminate viral latencies 0, I, II and III patterns. As it was previously described in tonsils from IM patients, EBV latency profiles were characterized exclusively by LMP1 and EBNA2 expression. Kurth *et al*. described that LIII was prevalent^9^ together with a variable percentage of cells displaying a LI or LII profile^21^. On the other hand, LII pattern was observed in tonsillar GC B-cells from adult EBV carriers^22^, though LI was described to be expressed in non-IM adult tonsils, which is characteristic of either differentiating GC B-cells or differentiated memory B-cells^18^. According to this, we also defined latencies based on LMP1 and EBNA2 expression, and found exclusively LII or III in our series. Both LMP1 and EBNA2 presence without LMP2A and EBNA3A additional expression, respectively, were reported in EBV+ Diffuse Large B cell Lymphoma by Cohen *et al*.^23^,^24^. Remarkably, LII was statistically associated with non-GC region, in disagreement with the suggestion that both LMP1 and/or LMP2A proteins collaborate with B-cells through GC transit. This could suppose two alternatives in the present series of pediatric carriers: LII pattern was exclusively located at the IF region, or otherwise, lymphocytes with LII expression left the GC region without down-regulating antigen expression in their process to become a memory B-cell.

In younger children, we found that presence of viral antigens is preferentially restricted to subepithelial lymphocytes, mainly in those patients displaying LIII pattern and LMP1+ lymphocytes. Conversely, older patients exhibited viral antigen presence particularly in epithelial cells. Viral infection of epithelial cells *in vitro* has been described, demonstrating that EBV derived from epithelial cells increases its ability to infect a B-cell, while EBV originated from B-cells improves its ability to infect epithelial cells^25^. Therefore, it is conceivable that in our series the subepithelial lymphocytes with a LIII expression profile associated with young ages and hence, might reflect an initial or recent infection. In contrast, older patients could probably have virus with increased tropism for epithelial cells, resulting from a past infection in the surrounding B-cells. This could be suitable to eventually shed virus into saliva and spread it to new hosts.

EBV viral load in tonsils was assessed to verify whether it was associated with a specific latent or lytic antigen expression. In studies from adult patients, viral load values in acute IM are 100-fold higher than in long-term carriers^26^. Previously, our group described in pediatric IM at onset of symptoms, variable levels of EBV genome, but still much lower than in PTLD or immunocompromised patients^27^. Therefore, low viral loads in tonsil samples from our pediatric series were not unexpected. In fact, given that the amount of EBV in tonsils may dictate the amount of virus shed in saliva^28^, low viral load confirms the assumption that the small inoculum influences whether primary EBV infection will be silent, as observed in our population, or will manifest as IM^29^. In addition, this was reinforced by the lack of association between lytic antigen BMRF1 expression and viral load. Concerning this issue, EBV may also express a limited ‘abortive’ form of lytic infection, where gene expression is limited to Zta and a few early viral proteins, which can also explain the low viral loads depicted^30^. Remarkably, the finding that the median viral load was higher specifically in cases with LMP1+ subepithelial cells reinforces the idea that recent infection in subepithelial lymphocytes is associated with higher viral load, and this could probably reflects recent viral infection.

Finally, since only 5 cases exhibited lytic BMRF1 antigen expression, lytic cycle might not denote a central role in our study of EBV pathogenesis in pediatric carriers. This could arise from a successful immune control of lytic viral cycle, and the subsequent low levels of productive infection in the asymptomatic condition. Efficient control of lytic EBV antigens in primary infection by NK cells was previously demonstrated in pediatric patients^16^. This type of cells could be involved in lytic antigen down-regulation in the present series. In addition, Chaganti *et al*. found occasional BZLF1-positive cells in IM tonsils but there was no clear evidence of cells completing lytic cycle^19^.

EBNA3A and 3C proteins are known to promote proliferation and are necessary for *in vitro* immortalization^31^. EBNA3A protein expression was not previously studied, either in IM patients or in healthy carriers. EBNA3A+ lymphocytes were detected without a specific age association or distribution. This fact points out that, in some degree, this antigen could strengthen the oncogenic mechanisms of the other EBV latent proteins and hence contribute to the pathogenesis of pediatric EBV-associated lymphomas. The unexpected observation of EBNA3A specific staining exclusively in subepithelial macrophages detected in some cases could reflect a successful phagocytosis of this antigen. In fact, EBV latent-cycle proteins display a marked hierarchy of immunodominance for CD8+ T-cell response (EBNA3C > EBNA1 > LMP2 ≫ LMP1)^32^. Moreover, viral antigen detection in the cytoplasm of macrophages due to effective phagocytosis was already described in viral infections^33^. Macrophages moving toward the EBV infected cells followed by their phagocytosis were also described in Burkitt lymphoma^34^. Surprisingly, the presence of EBNA3A in macrophages together with EBNA3A negative lymphocytes was associated with older patients, and could indicate that their immune response is effective rendering the down-regulation of EBNA3A, with the corresponding lack of this antigen in B-cells. In contrast, in young children the still immature immune system might not yet be able to control EBNA3A expression, giving the detection of EBNA3A+ lymphocytes.

Given that LIII pattern prevailed in our series and based on the known oncogenic potential of EBV *in vitro*, we can propose that the synergism of oncogenic viral proteins could occur in these cases. This finding, together with prior epidemiologic observations of higher prevalence of EBV-associated lymphomas in our pediatric population^17^ suggests that there could be an implication of EBV infection involved in B-cell lymphoma development in our country.

In summary, in our pediatric series, viral antigens were expressed at subepithelial lymphocytes at younger age, denoting that probably the virus has recently entered and it was allowed to express the full latent antigen profile, namely LIII. While, in older patients, EBV presence was detected mainly in epithelial cells, perhaps because they were infected a long time ago, and the virus has reached this location (epithelial cells) where it can spread out to another hosts. This finding is also sustained by viral load, which is higher in patients with cells expressing LMP1 at the SubEp region. In addition, we found that the proposed EBV infection models were not mutually exclusive in our population, since evidences from both infection of naïve B cells (GC model) and the location of LII outside GC (direct infection model), were observed. Finally, this analysis provides some basis to further understand EBV infection dynamics and try to explain more accurately EBV infection models in pediatric asymptomatic carriers from a developing country.

## Material and Methods

### Patients and samples

We studied 29 children’s formalin-fixed paraffin-embedded (FFPE) tonsil samples retrospectively selected from the archives of the Pathology Division at the Ricardo Gutierrez Children’s Hospital (Buenos Aires, Argentina). Cases displayed an age range of 2–14 years (median 5 years). Tonsil samples were surgically removed during routine tonsillectomy. Tonsillar hyperplasia was diagnosed according to international routine protocols.

Institutional guidelines regarding human experimentation were followed, and they were in accordance to the Helsinki Declaration of 1975. The Ethical Committee of the Ricardo Gutierrez Children’s Hospital (Buenos Aires, Argentina) has approved it and written informed consent was obtained from all patients’ parents.

### EBV detection by *in situ* hybridization

Specific infected cells together with the assessment of their histological location were determined by EBERs transcripts *in situ* hybridization (ISH) using PNA ISH Detection Kit (Dako), according to the manufacturer’s instructions.

### Immunohistochemistry

Immunohistochemistry (IHC) was performed to detect and localize EBV latent and lytic protein expression, using the following antibodies: LMP1 (mAb CS1-4, Dako); LMP2A (mAb 15F9, Abcam); EBNA2 (clones 1E6 and R3, kindly provided by Dr. Kremmer, Forschungszentrum fur Umwelt und Gesundheit GmbH, Institut fur Immulogie, Munchen, Germany); EBNA3A (Sheep polyclonal, Abcam); BMRF1 (mAb G3-E31, Abcam), as described by Cohen *et al*.^23^. The positive controls were performed in FFPE EBV+ cells lines (Raji and P3HR1, for EBNA2 and EBNA3A, respectively), P3HR1 treated with TPA (*12-O-tetradecanoylphorbol-13-acetate*, Sigma) to stimulate lytic infection (for BMRF1), FFPE EBV positive diffuse large B-cell lymphoma (for EBERs) and Hodgkin Lymphoma (for LMP1 and 2A). As negative controls we tested the specific primary antibody in FFPE tonsils from EBV sero-negative patients and also performed the same method without the primary antibody. This set of antibodies allowed us to establish the EBV latency profile.

### EBV viral load by real-time PCR

Viral load was determined in cases that had good quality DNA. The single copy gene BALF5 which encodes viral polymerase was amplified as described previously by Wadowsky *et al*.^35^.

### Double immunostaining

#### EBERs+IgD+ cells detection

ISH was performed as previously described and the same sections were subsequently subjected to antigen retrieval with 20 mM Tris/0.65 mM EDTA buffer pH 8 at 121 °C for 5 min. Then sections were allowed to cool at room temperature for 20 min, and rinsed in buffer. After blocking with avidin and biotin (Biotin Blocking System Dako-Cytomation, Carpinteria, CA), sections were incubated with anti-IgD monoclonal antibody (NCL-L-IgD, Novocastra, clone DNR1C, 1/1000) for 30 min. Then a streptavidin-biotin-peroxidase complex detection system (PolyTek HRP Anti-Mouse Polymerized Imaging System, Scytek) was used according to the manufacturer’s instructions.

#### EBERs+CD27+ cells detection

Was performed as described above for EBERs-IgD+ cells, but the antigen retrieval solution used was 1 mM EDTA buffer pH 8 at 121 °C for 5 min. Anti-CD27monoclonal antibody (NCL-CD27, Novocastra, clone 137B4, 1/80) was used and detection was performed using a goat anti-mouse IgG FITC conjugated (ab6785) (1:1000) ON at 4 °C. Finally, sections were stained with nuclear dye Höechst, counterstained with Evans Blue and covered using commercial aqueous mounting medium. As detection of EBERs was done using an alkaline phosphatase system and NBT as substrate, images were converted to a false red color with the Image-Pro Plus software version 6.0.0.260 and then merged with fluorescent ones.

#### LMP2A+cytokeratin+ cells detection

It was performed in order to demonstrate EBV latent antigen expression in epithelial cells. IHC for LMP2A was assessed as previously described, but LMP2A primary Ab was detected with rabbit anti-rat IgG FITC conjugated (Vector Labs). Subsequently, the same sections were subjected to antigen retrieval with 20 mM Tris/0.65 mM EDTA buffer pH 8 at 121 °C for 5 min. After blocking, sections were incubated with rabbit monoclonal anti-Cytokeratin 7 Ab (Ventana), and detected with goat anti-rabbit IgG (AlexaFluor®568, ab175471, Abcam) as secondary Ab. Finally, sections were stained with nuclear dye Höechst and covered using commercial aqueous mounting medium.

### Statistical analysis

Statistical analysis was performed using GraphPad Prism 5 and InStat 3 software (GraphPad Software Inc., San Diego California USA). Categorical variables were analyzed using Fisher’s exact test. Mann–Whitney test was used to compare the means between age groups or viral load in relation to EBV presence or type of EBV-infected cells. All tests were two-tailed, and p < 0.05 was considered statistically significant.

## Additional Information

**How to cite this article**: Vistarop, A. G. *et al*. Analysis of Epstein-Barr virus infection models in a series of pediatric carriers from a developing country. *Sci. Rep*. **6**, 23303; doi: 10.1038/srep23303 (2016).

## Figures and Tables

**Table 1:** Description of EBV protein expression in tonsil sections from pediatrics carriers.

Latencies patterns	Latency Location	Age	ISH EBERS	Location	Viral Load #copy/μg/μl	Protein Expression										Epithelium	Double Staining	
						LMP1	Location	LMP2A	Location	BMRF1	Location	EBNA3A	Location	EBNA2	Location		EBERS-CD27	EBERS-IgD
III	IF-SubEp	3	+	IF	30	+	IF	−		−		+	IF-SubEp	+	IF	−	−	ND
III	IF-SubEp	5	+	IF	30	+	IF	+	IF	−		+	IF	+	IF-SubEp	−	−	ND
III	IF	3	+	IF	ND	+	IF	−		−		−		+	IF	−	ISH −	−
III	IF-SubEp	7	+	IF	172	+	IF	−		−		−	M-P	+	SubEp	+	−	+
III	IF-GC-SubEp	2	+	IF-GC	185	+	IF-SubEp	−		−		+	IF	+	IF-GC	−	−	+
III	IF-GC-SubEp	3	+	IF-GC	119	+	IF-SubEp-M	+	IF	−		+	ND	+	IF-GC	−	+	+
III	IF-GC-SubEp	12	+	IF	60	+	IF	+	IF-P	−		−	M	+	GC-SubEp	+	ND	+
III	IF-GC-SubEp	4	+	IF	60	+	IF	+	IF-SubEp	−		−		+	IF-GC	−	−	+
III	IF-GC-SubEp	5	+	IF	60	+	IF	−		−		+	GC	+	IF-SubEp	−	−	+
III	IF-GC-SubEp	5	+	IF-GC	60	+	IF-CG	−		−		+	SubEp	+	GC-M-L	−	−	+
III	IF-GC-SubEp	6	+	IF	ND	+	IF-SubEp-M	+	GC	+	near muscle	+	IF	+	GC	−	ND	−
III	IF-GC	10	+	IF	ND	+	IF	+	IF-GC	−		−	M	+	GC-M-L	−	−	+
III	IF-GC	14	+	IF	30	+	IF	+	IF-P	−		−	M	+	GC	+	−	+
III	IF-GC	13	+	IF	30	+	IF	+	IF	−		+	IF-P	+	IF-GC-P	+	−	ND
III	IF-GC	9	+	IF	ND	+	IF-M	+	IF-GC-P	−		−	P	+	IF-GC	+	ND	−
III	IF-GC	5	+	IF	ND	+	IF	+	IF-P	−		−		+	GC	+	−	+
III	IF-GC	12	+	IF	60	+	IF-M	+	IF	−		−	M	+	IF-GC	−	−	−
III	IF-GC	6	+	IF	60	+	IF-M	−		−		+	IF-P	+	IF-GC	+	−	−
III	IF-GC	9	+	IF-GC	60	+	IF	−		+	IF-P	+	IF-GC	+	IF	+	+	+
II	IF-SubEp	3	+	IF	30	+	IF-SubEp	+	IF	−		−		−		−	+	−
II	IF-SubEp	2	+	IF	60	+	IF-SubEp	+	IF-SubEp	−		−		−		−	−	+
II	IF	8	+	IF	60	+	IF	+	IF	−		−		−		−	−	−
II	IF	7	+	IF	ND	+	IF	−		−		−		−		−	ND	−
II	IF	4	+	IF	60	+	IF	+	IF-P	−		−		−		+	ND	ND
II	IF	2	+	IF	372	+	IF	−		+	IF	−		−	P	+	+	+
II	IF-GC-SubEp	5	+	GC	153	+	IF-SubEp	+	IF-P	−		−		−		+	ND	+
II	IF-GC-SubEp	6	+	GC	ND	+	IF-SubEp	+	IF-P	−		−	M	−		+	−	−
II	IF-GC	6	+	IF	30	+	IF	+	IF-GC-P	+	IF	−		−		+	−	−
II	IF-GC	2	+	IF-GC	60	+	IF-M	+	IF	+	IF	−		−		−	−	+

EBV protein expression in each EBV+ sample listed according latency pattern; histological characteristics; latency location; viral load; naïve (IgD), memory and/or not-switch (CD27) population double staining. EBERs ISH indicates EBV-encoded RNAs by *in situ* hybridization; LMP1, latent membrane proteins 1; LMP2A, latent membrane proteins 2A; BMRF1, EBV early lytic protein; EBNA3A, Epstein Barr nuclear antigen 3A; EBNA2, Epstein Barr nuclear antigen 2A; IF, interfollicular region; SubEp, subepithelial region; GC, germinal center region; P, epithelium; M, macrophages; L, lymphocyte; ND, not determined.
